# Supplementary material for: Empowering Patients to Self-Manage Common Infections: Qualitative Study Informing the Development of an Evidence-Based Patient Information Leaflet
Source: Antibiotics (Basel). 2021 Sep 15;10(9):1113. doi: 10.3390/antibiotics10091113 (PMC8464808; doi:10.3390/antibiotics10091113)
Supplement: Supplementary file 1 [file antibiotics-10-01113-s001.zip › Supplementary S1 patient and HCP interview schedules.pdf]

antibiotics

MDPI

Article

Empowering Patients to Self-Manage Common Infections: Qualitative Study Informing the Development of an Evidence-based Patient Information Leaflet

Catherine V. Hayes *, Bláthnaid Mahon, Eirwen Sides, Rosie Allison, Donna M. Lecky and Cliodna A. M. McNulty

Primary Care and Interventions Unit, Public Health England, Gloucester GL1 1DQ, UK; Blathnaid.mahon@phe.gov.uk (B.M.); Eirwen.sides@phe.gov.uk (E.S.); Rosie.allison@phe.gov.uk (R.A.); Donna.lecky@phe.gov.uk (D.M.L.); Cliodna.mcnulty@phe.gov.uk (C.A.M.)
* Correspondence: Catherine.hayes@phe.gov.uk

1. Patient interview schedule

Introduction *[please read this to the interviewee before the interview takes place]*

My name is XXX, and I am interviewing you on behalf of Public Health England to understand what people do when they feel unwell or have a common uncomplicated infection. **When I say a common minor infection I mean: An infection that usually gets better on its own without any specific antibiotic, or only a very short course of simple antibiotics and has no long-term harmful effect on a person's health (assuming that they are otherwise healthy). Examples include most sore throats, colds, flu, oral thrush and winter vomiting bugs and some UTIs.**
(https://www.nice.org.uk/guidance/qs121/chapter/Quality-statement-1-Advice-on-self-limiting-conditions)

The information you give us will be used to help develop a patient information self-care leaflet covering common infections. This leaflet will be used by healthcare staff in community pharmacies when they dispense antibiotics or give general self-care advice. It could also be used in other settings.
I would like to ask you about three topics.

- Firstly, I would like to talk to you about what happened and what you did when you were unwell.
- Secondly, if there was an instance when you asked for or were given infection related advice at a community pharmacy, what was your experience?
- Finally, I will ask for your feedback on patient information we are developing.

If you don't mind, the interview will be recorded and I will take a few notes.The notes and recording will be anonymised before we type it up, meaning we will not use your name or any other information that could be used to identify you. Are you happy to go ahead with the interview? Can I check that you have signed the consent form?
If you have anything that you would like to add that you have not thought of during the interview, please email the research team.

Section One –

1. Tell me about what you do to stay healthy and infection-free? *Probe: Stay hydrated, exercise, eat healthy, wash hands at appropriate time etc…* (knowledge) (skills) probe – was this different before COVID-19? Can you think back to last year? What do you think you will be doing going forward?

2. Can you tell me a little bit about the last time you felt unwell with an infection? *Probe: How long did it last? Did you self-care? Take antibiotics? Seek advice?*
3. What sort of things do you do to try to help your symptoms, if at all? *Probe: drink more liquids? Rest? Take paracetamol/ibuprofen or other pain relief? OTC/other products?*
4. To what extent are you confident in managing your own symptoms when you are unwell? (beliefs about capabilities) (optimism) probe what gives you the confidence and skills -
5. How would you obtain advice when you feel unwell? *Probe: Friends, family, carers, nurse, doctor, pharmacist?* Internet and which sites Instagram etc(Environmental context and resources) (Memory, attention and decision making)

   Who is your first port of call? *Probe family, friends*

Section Two –

6. Can we discuss what you do next when you feel you are getting worse and need more help or advice?
7. At what point would you seek health care advice when you are unwell? *Probe: from who? pharmacist, GP? Type of symptoms?* (Memory, attention and decision making)
   a) Can you tell me about this process? *Probe: what happened the last time you sought professional advice when you had a self-limiting infection?*
8. Can you tell me about the last time you asked for advice at a community pharmacy?
   a. What sort of common infections would you be happy to ask about at a pharmacy
9. How do you feel about discussing your symptoms in a community pharmacy? *Probe: who would you prefer? pharmacist, , counter staff. Is there someone you definitely wouldn't discuss this with?* (emotion)
10. What outcome are you looking for when you discuss your symptoms with a community pharmacist? *Probe: self-care advice, OTC products, referral?* (beliefs about consequences) (goals)
    a. How would this differ from your expectation from a / GP / nurse?
11. Would you do anything different or expect anything to be done differently if caring for a child or an elderly person? (Memory, attention and decision making)
12. Can you tell me about any information that, pharmacists and other pharmacy staff. have shared with you around how to look after yourself when you feel unwell? *Probe: Information, safety netting*
    a) Who gave you the information?
    b) What questions did they asked you?
    c) Was their advice clear, did you understand?
    d) Did they you give you any advice on specific symptoms to look out for which would indicate that you need to seek further medical advice/assistance? *Probe: what symptoms, who did they say to contact/go to?*
    e) Is there anything that you think they should have told you or wish they had done differently? *Probe: Is there anything from your perspective that you would like clinicians to know?*
    f) *How was this information given – in the form of a leaflet, did they share the leaflet or did they refer you to other information*
    g) *What do you think about being given a QR code to download information onto your phone or tablet?*

13. Has anyone ever given you any type of leaflet/information about self-care? *Probe: Leaflet, posters, booklets, electronic information etc.* (environmental context and resources)
14. What kind of information, if any, would you like to receive when you are unwell? Probe: what sort of information would be useful when you pick up an antibiotic prescription – has this occurred when you have picked up an antibiotic prescription for yourself or others recently? *Probe seek when to seek further advice/assistance, self-care, AMR.* (behavioural regulation)
15. How would it be best to receive this information? *Probe: leaflets, posters, verbally, electronic information?* (behavioural regulation)
16. What are the priority areas for support? *Probe: condition, self-care advice, safely netting, AMR explanation* (Environmental context and resources)
17. Would you use a resource like this leaflet if we were to develop it? (Intention)
18. What would encourage you and others to use this resource? (reinforcement)

-BREAK-

Section Three – leaflet feedback

19. This patient information self-care leaflet is currently being developed. May we review each section? *(Here, show the participant the leaflet – ask participant to think aloud their thoughts on the content)*

Probes:

a. The things you like about the way the leaflet looks. *Probe: Colour, layout, font*
b. What don't you like about the way the leaflet looks? *Probe: Colour, layout, font*
c. Can you think of any suggestions for the way the leaflet should look? *Probe: more pictures, bigger text, smaller sentences, more diagrams, two-sided, tri-fold etc.*
d. What do you think are the most important sections for you? *Probe: most useful, most interesting, new information, crucial information*
e. What are the least important sections, if any? *Probe: Not relevant, too complicated, don't need to know*
f. What do you think of the wording used in the leaflet? *Probe: Is it appropriate for patients? Pictorial representations? What about those with cognitive disabilities?*

Other questions:

20. How Do you think you would be able to manage your symptoms at home (if at all) based on the information in the leaflet?
21. How could you keep this leaflet so that you have it to hand when you need to refer to it in the future?
22. How do you think this leaflet could be used in the community or hospital pharmacy setting?
23. Is it the sort of thing that you would be happy to receive if picking up an antibiotic prescription for another family member or friend ?
24. Do you think the leaflet reinforces things that you already do – or is there new information there? If so please do highlight these

25. Can you foresee any difficulties in using such a resource? *Probe: follow up on issues raised.*
26. Do you have any other thoughts or comments that I haven't touched upon?

2. Interview schedule for healthcare professionals

Introduction *[please read this to the interviewee before the interview takes place]*
My name is XXX, and I am interviewing you on behalf of Public Health England, to understand your attitudes and experiences of managing patients with common infections so that we can broaden our patient facing resources .
The information you give us will be used to help develop a general patient facing leafletcovering the self-care and safety-netting of common infections. The leaflet will most commonly be used by staff dispensing antibiotics or giving general advice when the exact cause of possible infection related symptoms is not known.

I would like to ask you about three topics.

- Firstly, I would like to talk to you about ways in which you manage patients with common infections and your experiences with this.
- Secondly, I would like to ask you about potential tools/resources to support you and other healthcare professionals in providing self-care and safety-netting advice to patients, including implementation and any potential barriers.
- Finally, I would like to ask you about how you think the self-care leaflet might be improved and whether you think there are any problems with using such a resource.

If you don't mind, the interview will be recorded and I will take a few notes.The notes and recording will be anonymised before we type it up, meaning we will not use your name or any other information that could be used to identify you. Are you happy to go ahead with the interview? Can I check that you have signed the consent form?

If you have anything that you would like to add that you have not thought of during the interview, please email the research team
Section One –

1. How would you define a common infection? *Probe: Can you name any*? (Knowledge)

   At this point establish their terminology for a common infection and ensure use of the term throughout the interview.

   When I say common infection I mean: A condition that resolves on its own and has no long-term harmful effect on a person's health (assuming that they are not immunosuppressed). Examples include sore throats, colds, flu, some urinary symptoms and winter vomiting bugs. (https://www.nice.org.uk/guidance/qs121/chapter/Quality-statement-1-Advice-on-self-limiting-conditions)

2. Can you talk me through how you would manage a patient with a common infection? *Probe: OTC* (skills)
3. can you tell me about the value (if any) to you and patients of giving self-care advice? *Probe: effectiveness, leads to faster recovery, prevention, hygiene and transmission to others?* (Beliefs about consequences)
4. tell me about how you fit (if at all) self-care advice into the consultation process?
   a) Can you tell me about the most common self-care advice you give to help them recover faster? (memory attention and decision making) - probe rest, fluids, pain relief, specific remedies for different infections

b) What sort of safety netting advice would you usually give? Probe: symptoms, where/when to seek further assistance (memory attention and decision making).

c) Tell be about any common barriers you experience in giving self-care and safety netting advice during the consultation process? Probe: patient factors, personal factors time, opportunity, memory forgetfulness, language

5. How confident are you in providing self-care advice to patients/carers? *Probe: Any conditions that pose more of a problem? Easier conditions to advise on?* (Beliefs about capabilities, also could probe about if they think they have the knowledge to cover these things)

6. How confident are you in giving safety-netting advice to someone with a common infection? (Beliefs about capabilities)

7. In your particular work setting, tell me about the different staff members roles in managing patients with common infections? *Probe: advising, sign posting etc. (Professional roles and identity)*

8. Tell me about any processes in place (if any) to ensure all staff give consistent self-care advice? *Probe: training* (Memory, attention and decision making)

9. Tell be about how you consider patients/carers listen and act upon your advice? *Probe: their knowledge, is there an emotional component, confidence in providing self-care advice as opposed to antibiotics* (Social influence & Emotion)

   a) tell me about clients (for pharmacists) / patients (for GP staff) attitudes that you have encountered when giving self-care advice? *Probe: How satisfied are patients and their carers with the self-care advice you give?* (Social influence)

10. Tell me about any processes in place in your setting to monitor or regulate delivery of self-care and safety netting advice? *Probe: audits, checklists, reminders, guidelines (Behavioural regulations)*

11. tell me about how patients raise the topic (if at all) of antibiotics during a conversation about common infections? (Skills/interpersonal skills)

    a) How do you respond when this happens?

12. can you tell me a bit about when and how you raise the topic (if at all) of antibiotic resistance with clients/patients? (Motivation and goals)

13. Can you tell me a bit about how antibiotic resistance influences you on a day to day basis, if at all? *Probe: Do you have an example of a resistant infection?* (Behavioural regulation)

14. how do you think appropriate antibiotics for minor infection can help the development of resistance, if at all? (Beliefs about consequences)

    a) How optimistic are you (if at all) that you can make a difference to antibiotic resistance? (Optimism)

15. How do you think you could help reduce the number of patients who are prescribed inappropriate antibiotics for a common infection? (Behavioural regulation)

Section Two

16. Can you tell me about any resources/tools you use (if any) to assist you in advising patients with minor common infections? *Probe: leaflets, guidelines, etc.* (Environmental context and resources)

17. Could you reflect on what kind of support would help healthcare professionals in advising patients with minor infections? *Probe: Information/guidance/leaflet/app (Skills/interpersonal skills)* probe: priority areas for support? *Probe: condition, self-care advice, safely netting, AMR explanation* (Environmental context and resources)
18. could you reflect on how you might (if at all) use a patient facing resource if we were to publish it? (Intention)
19. What sort of national or local initiatives might encourage healthcare professionals in pharmacies to use this resource? (reinforcement) probe – would you get involved in them

Section Three –

20. This patient information self-care leaflet is currently being developed. May we review each section? *(Here, show the participant the leaflet – ask participant to think aloud their thoughts on the content)*

Probes:

a. The things you like about the way the leaflet looks. *Probe: Colour, layout, font*
b. What don't you like about the way the leaflet looks? *Probe: Colour, layout, font*
c. Can you think of any suggestions for the way the leaflet should look? *Probe: more pictures, bigger text, smaller sentences, more diagrams, two-sided, tri-fold etc.*
d. What do you think are the most important sections for you? *Probe: most useful, most interesting, new information, crucial information*
e. What are the least important sections, if any? *Probe: Not relevant, too complicated, don't need to know*
f. What do you think of the wording used in the leaflet? *Probe: Is it appropriate for patients? Pictorial representations? What about those with cognitive disabilities?*

21. Thinking specifically about the information on the leaflet, can you tell me:

a. What do you think are the most important sections for you? *Probe: most useful, most interesting, new information, crucial information*
b. What are the least important sections, if any? *Probe: Not relevant, too complicated, don't need to know*
c. What do you think of the wording used in the leaflet? *Probe: Is it appropriate for patients? Pictorial representations? What about those with cognitive disabilities?*

22. Do you think patients would use this leaflet?
23. How (if at all) Do you think a leaflet like this could help patients prevent and manage minor infections? If so, how? *Probe: prevent future infections; give patients the confidence to look after their own UTI?*
24. If we changed this leaflet in line with your suggestions do you think patients would use it, and why?
25. what sorts of barriers may potential users come across when using such a resource? *Probe: follow up on issues raised.*
26. Do you have any other thoughts or comments that I haven't touched upon?
