# Supplementary material for: Empowering Patients to Self-Manage Common Infections: Qualitative Study Informing the Development of an Evidence-Based Patient Information Leaflet
Source: Antibiotics (Basel). 2021 Sep 15;10(9):1113. doi: 10.3390/antibiotics10091113 (PMC8464808; doi:10.3390/antibiotics10091113)
Supplement: Supplementary file 1 [file antibiotics-10-01113-s001.zip › Supplementary S2 HCP and minority ethnic patient questionnaires.pdf]

antibiotics

MDPI

*Article*

# Empowering Patients to Self-Manage Common Infections: Qualitative Study Informing the Development of an Evidence-based Patient Information Leaflet

**Catherine V. Hayes *, Bláthnaid Mahon, Eirwen Sides, Rosie Allison, Donna M. Lecky and Cliodna A. M. McNulty**

Primary Care and Interventions Unit, Public Health England, Gloucester GL1 1DQ, UK; Blathnaid.mahon@phe.gov.uk (B.M.); Eirwen.sides@phe.gov.uk (E.S.); Rosie.allison@phe.gov.uk (R.A.); Donna.lecky@phe.gov.uk (D.M.L.); Cliodna.mcnulty@phe.gov.uk (C.A.M.)

* Correspondence: Catherine.hayes@phe.gov.uk

## Survey 1 for health professional feedback on leaflet

**Page 1: Introduction and about you**

This survey is really important as it allows you to optimise a new TARGET 'managing your infection' leaflet aimed at improving self-care and safety netting advice to patients and reflect on your own practice. This can be used for continuing professional development.

**Any questions please contact XXXX**

*1. What is your professional role?* (open)

*2. What organisation do you work for?* (open)

*3. Does your role include providing self-care or other infection related advice to patients?* (Yes/no)

**Page 2: First page of leaflet**

You will be shown each page of the 4-page leaflet which will fold into an A5 booklet, please provide comments on the page shown to you. There will also be opportunity to provide overall feedback on the whole leaflet.

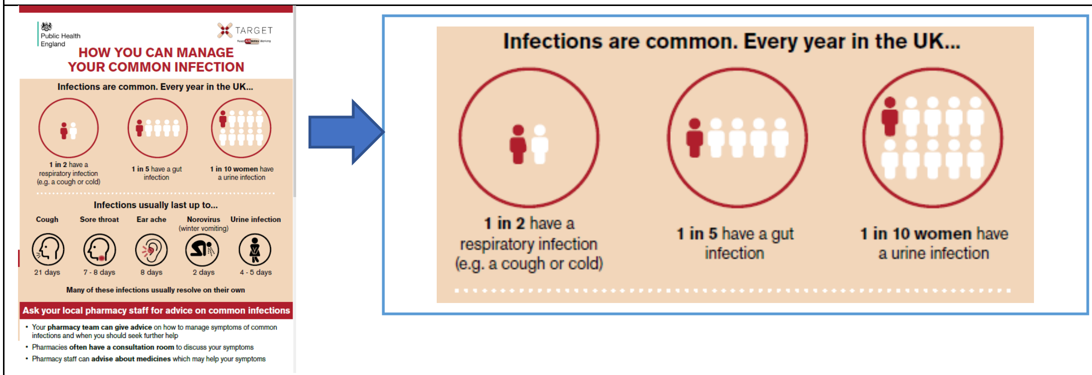


*The image above is from the first page of the leaflet*

*4. On a scale of 1-5 how useful is this section to patients*
*Why and please suggest any changes (open)*

5

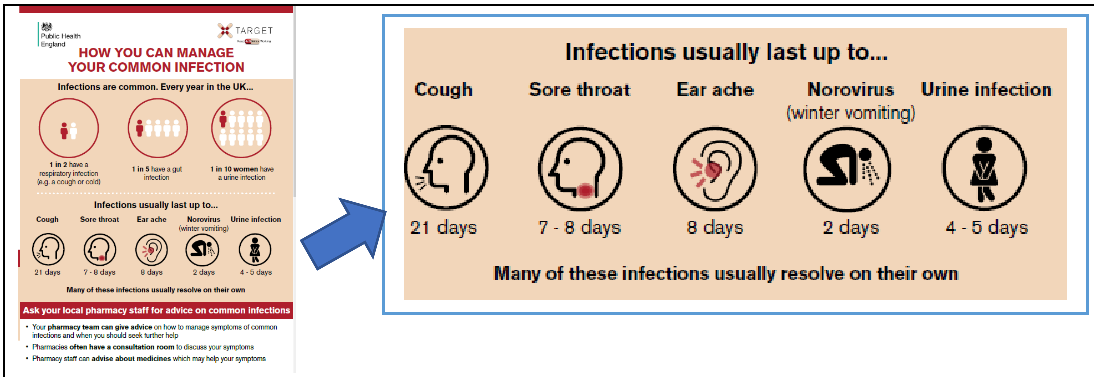


*The image above is from the first page of the leaflet*

*On a scale of 1-5 how useful is this section to patients?*
*Why? please comment on the design and ease of sharing with patients (open)*
*Considering this is a general infection leaflet for healthcare staff to give to patients, are there other important omissions that usually resolve on their own? (open)*

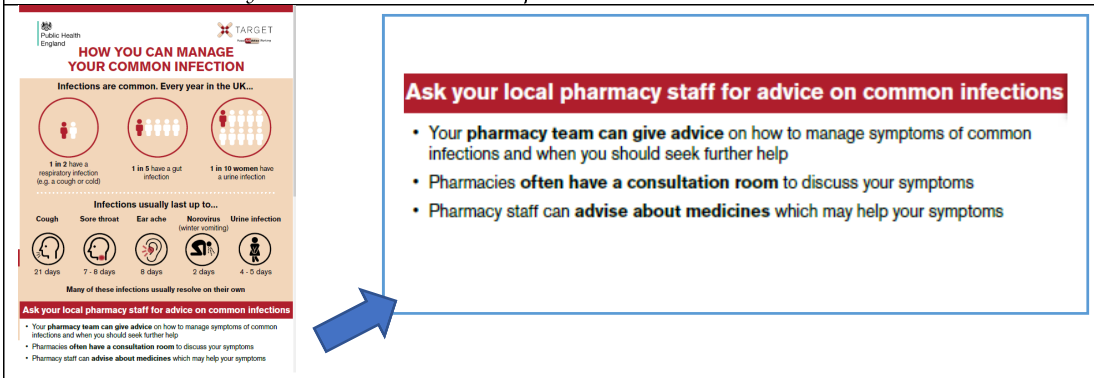


*The image above is from the bottom of the first page of the leaflet*

*6.how useful on a scale 1-5 do you consider this section is to patients*
*Why? please comment on the wording and ease of sharing with patients (open)*

*Considering this is a general infection leaflet for healthcare staff to give to patients, and it needs to be simple is there anything else that should be included here? Please note that there is advice on when to seek urgent help on the back page.*

**Page 3: Middle spread of leaflet**

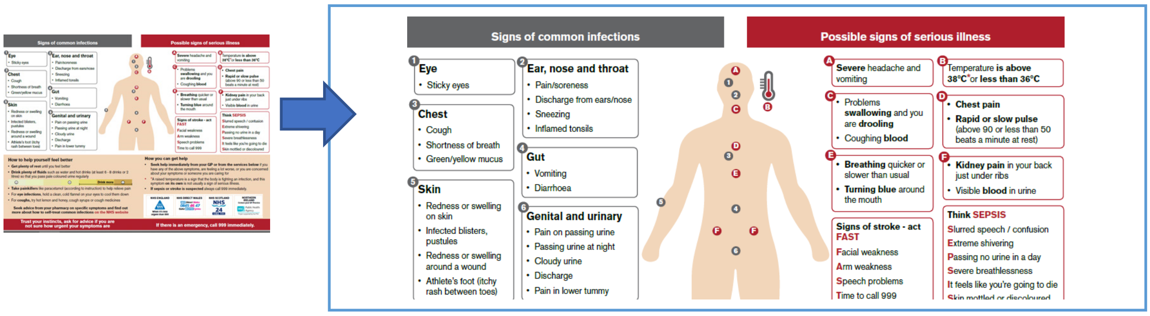


The image above is from the middle spread of the leaflet (page 2 and 3).

*7. how useful on a scale 1-5 do you consider this section with common signs and symptoms on one side and signs of serious illness on the other is to patient?*
*Why? Please state if there is something you particularly like or dislike and ease of sharing with patients (open)*
*Do you think the numbering and lettering is clear – how (if at all) could it be improved*
*Are there any words we should remove or change? (open)*

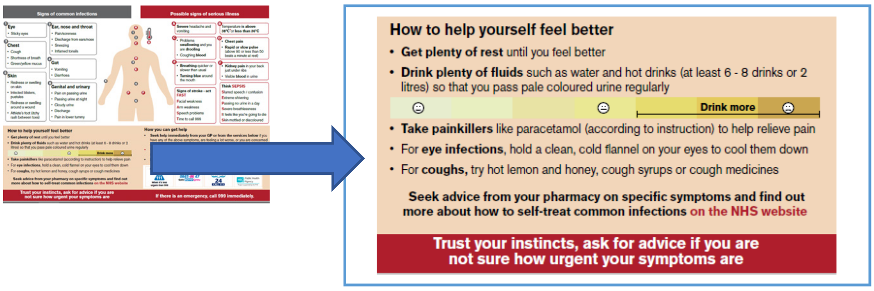


The image above is from page 2 of the leaflet . How to help yourself get better

*9. how useful on a scale 1-5 is the self-care advice to patients*
*Please state why, and anything you particularly like or dislike? (open)*
*is there any wording we should remove or change? (open)*
*Considering this is a general infection leaflet – is there any simple advice we should add? (open)*

*10. how useful on a scale 1-5 is the wording to seek advice from your pharmacy*
*Why and please suggest any changes to wording (open)*

*11. how useful on a scale 1-5 is the red bottom box to trust your instincts*
*Why and please suggest any changes to wording (open)*

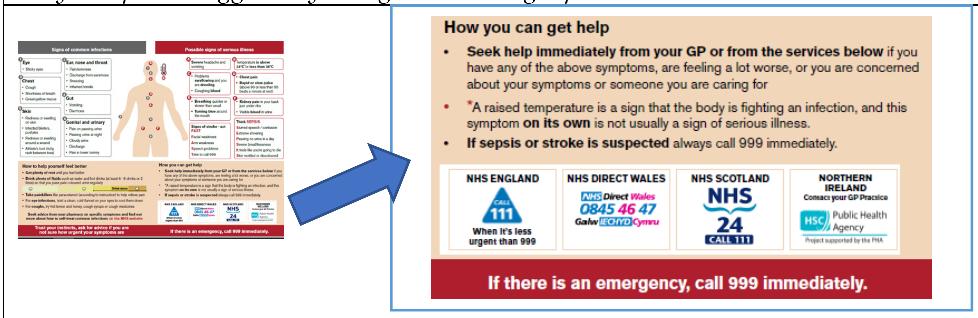


The image above is from the page 3 of the leaflet (page 3).

*12.how useful on a scale 1-5 is the "How you can get help" advice to patients*
*Please state why, and anything you particularly like or dislike? (open)*
*is there any wording we should remove or change open? (open)*
*Considering this is a general infection leaflet – is there any simple advice we should add? (open)*

*13. how useful on a scale 1-5 are the logos?*
*Please state why, and any that should be removed or changed (open)*

*14. how useful on a scale 1-5 is the red box "if there is an emergency, call 999 immediately"*
*Please state why, and wording that should be removed or changed (open)*

**Page 4: back page of leaflet**

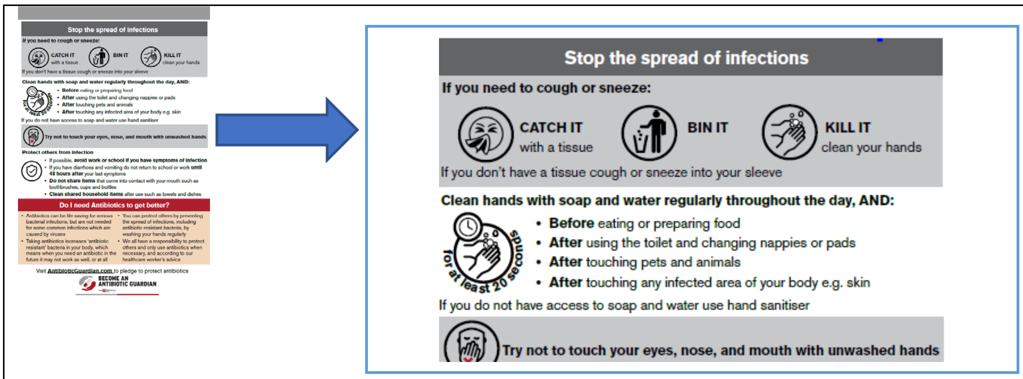


*The image above is from "stop the spread of infections" section of the back page of the leaflet (page 4).*

*15.how useful on a scale 1-5 is the "stop the spread of infections" advice to patients*
*Please state why, and anything you particularly like or dislike? (open)*
*is there any wording we should remove or change open? (open)*
*Considering this is a general infection leaflet – is there any simple advice we should add? (open)*

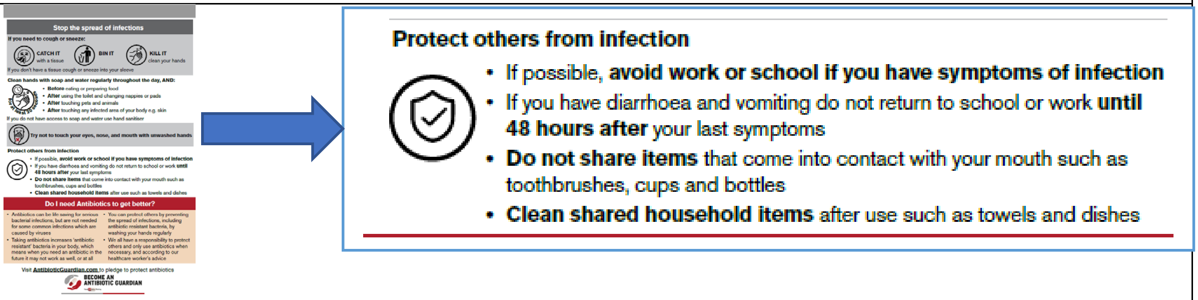


*The image above is from the "protect others from infection" section of the back page of the leaflet (page 4).*

*16.how useful on a scale 1-5 is the "protect others from infection" advice to patients*
*Please state why, and anything you particularly like or dislike? (open)*
*is there any wording we should remove or change open? (open)*
*Considering this is a general infection leaflet – is there any simple advice we should add? (open)*

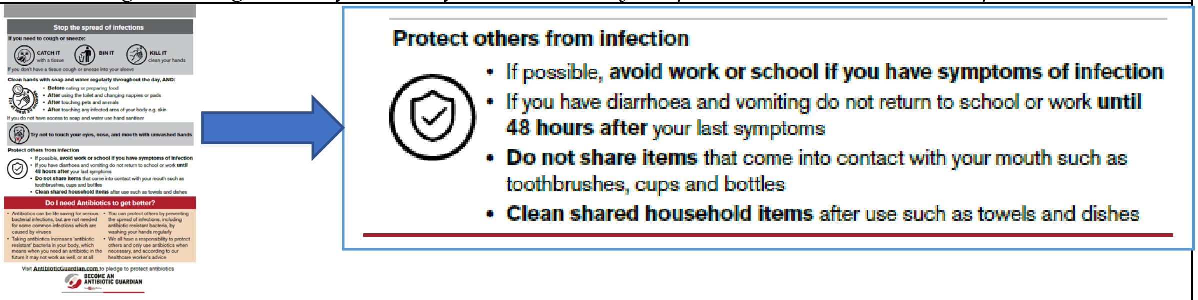


*The image above is from the "Do I need antibiotics to get better?" section of the back page of the leaflet (page 4).*

*17.how useful on a scale 1-5 is the "Do I need antibiotics to get better?" advice to patients*
*Please state why, and anything you particularly like or dislike? (open)*
*Is there any wording we should remove or change open? (open)*
*Considering this is a general infection leaflet – is there any simple advice we should add? (open)*

Visit AntibioticGuardian.com to pledge to protect antibiotics
BECOME AN ANTIBIOTIC GUARDIAN

**18.** *How useful on a scale 1-5 is the "link to the Antibiotic Guardian campaign?" advice to patients*
*Please state why,? (open)*

| |
|---|
| *Considering this is a general infection leaflet – is there any other link we would be better to use or add? (open)* |
| **Page 5: overall feedback on leaflet** |
| *7. What do you think of the wording used in the leaflet? (open)* |
| *8. Do you have any comments / suggestion on the leaflet aesthetics? (open)* |
| *9. How likely on a scale 1-5 are to you use this leaflet in your healthcare setting? (YES/NO)*<br><br>Follow up if answer 'no' – why? |
| *10. How likely on a scale 1-5 Do you think patients would use this leaflet? (YES/NO)*<br><br>Follow up if answer 'no' – why? |

## Survey 2 for minority ethnic patient feedback on leaflet

| |
|---|
| **Introduction**<br>We have developed this general information leaflet to help people identify and look after themselves when they have a common infection, including COVID-19.<br><br>We want to make sure this leaflet will be helpful for individuals from minority ethnic backgrounds. Your feedback on this leaflet will help us to support the health of minority ethnic communities.<br><br>Please answer the following questions from your perspective as an individual from a minority ethnic background. |
| 1. How useful is the overall content of this leaflet? (single option)<br>☐ Very useful<br>☐ Useful<br>☐ Neutral<br>☐ Useless<br>☐ Very useless<br><br>1a. Please tell us why you answered in this way |
| 2. In your opinion, does the leaflet provide all of the necessary information to help you look after yourself when you have a common infection? (single option)<br>☐ Yes<br>☐ No<br>If no,<br>a. What other information would you like to see in this leaflet? (Open)<br>b. What COVID-19 specific information would you like to see in this leaflet? (Open) |
| 3. Please describe the cultural factors of importance, if any that are missing from this leaflet (Open) |

| | |
|---|---|
| 4. | Please describe the barriers, if any, for individuals from minority ethnic groups in using this leaflet (Open) |
| 5. | Please describe the barriers, if any, for individuals from minority ethnic groups in doing any of the actions recommended in the leaflet (Open) |
| 6. | Please provide any further comments on this leaflet (Open) |
