# Supplementary material for: Empowering Patients to Self-Manage Common Infections: Qualitative Study Informing the Development of an Evidence-Based Patient Information Leaflet
Source: Antibiotics (Basel). 2021 Sep 15;10(9):1113. doi: 10.3390/antibiotics10091113 (PMC8464808; doi:10.3390/antibiotics10091113)
Supplement: Supplementary file 1 [file antibiotics-10-01113-s001.zip › Table S1.pdf]

antibiotics

MDPI

Article

Empowering Patients to Self-Manage Common Infections: Qualitative Study Informing the Development of an Evidence-based Patient Information Leaflet

Catherine V. Hayes *, Bláthnaid Mahon, Eirwen Sides, Rosie Allison, Donna M. Lecky and Cliodna A. M. McNulty

Primary Care and Interventions Unit, Public Health England, Gloucester GL1 1DQ, UK; Blathnaid.mahon@phe.gov.uk (B.M.); Eirwen.sides@phe.gov.uk (E.S.); Rosie.allison@phe.gov.uk (R.A.); Donna.lecky@phe.gov.uk (D.M.L.); Cliodna.mcnulty@phe.gov.uk (C.A.M.)

Table S1. Patient emerging themes on self-management of common infections (additional quotes).

Theme	TDF domain(s)	Sub Themes	Quotes
Preventing infections	Knowledge; Belief about consequences; Social influences; Reinforcement	Healthy lifestyle	'I eat as many, as much fruit and vegetables as possible and try and be as healthy as possible, dog walking, and running, and that kind of thing. I'm also at the moment just on, I'm taking a vitamin D supplement just because of the [COVID-19 pandemic] but normally I don't take any supplements. ' **(Female, FG5)**
		Motivations for preventing infections	'I've suffered from urinary tract infections so drinking plenty of water is really important for me and having a lot of vegetables and fruit' (**Female, FG5)**<br><br>'I go to the gym, I watch what I eat, I take some vitamins… I've got a couple of friends who died fairly early because of their lifestyle. Some people have no control over the disease they get but, and I think it brought it home to me so as I've got older it's, I'm more sensible with things. And as I say, it's a cliche and it's boring but everything in moderation really.' **(Male, FG2)**
		Social distancing and hygiene	'A lot of cleaning, a lot more cleaning. I used to clean a lot anyway but since this COVID-19, I find that it's just in my head all the time.' (**Female, FG4)**<br><br>'Self-quarantining, I think there's a little element of that, certainly with norovirus I would never go near anybody if I know I'm with someone who's got it or I'm going, know I'm going to get it. I've had it three times in my life now.' **(Female, FG1)**
Self-treating infections	Skills; Belief about consequences; Belief about capabilities; Environmental context	Self-care actions, attitudes and motivations	'Not being a child, you come across things that you've had in the past and you either using the tried and tested that you've done before… And if it is something that persists or goes on for longer, I go to the pharmacist.' **(Male, FG4)**<br><br>'I'll tell you one of the main things is not let it beat you. If you feel, if you've got a cold, whatever, just I like to carry on and keep doing with what I'm doing and not let it get to me and that seems to be the best way of dealing with it.' **(Male, FG2)**<br><br>**'Male participant 1**: But generally, with something like that, it's if I need it, *Lemsips*, stick with my mum's recipe of Honey and Lemon, which goes down a treat but generally as a rule with colds I'm poorly for about two days, and then, *that'll* be from, from the start to finish in two days and it's all over and done with, which annoys the hell out of people

			because they're there for a week or so *because they're being* ill, and I'm there getting over it quite quickly.<br>**Male participant 2**: If I've got a cold I just take a couple of paracetamol it seems to knock it back, I'd say…<br>**Female participant:** A couple of other things that I swear by that probably aren't general advice are things like a saline nasal spray and steam inhalation –'<br>**(FG3)**<br><br>'I'm a believer that if you start taking medication when you don't need to it loses its effect when you actually do need it…So, I try and stay off of it. But there, when I have had colds I usually go for like a Day and Night Nurse probably just to whack me out and control my temperature a little bit, I know it's probably just paracetamol that's in it really, but just to rest and that's basically all I do. I don't really take throat sweets or not cough medicine unless it's really uncomfortable, just try and get over it by myself really.'<br>**(Female, FG1)**
		Perceived skills and confidence	'Something much more serious and how do you know, where's the little line that you cross between self care and feeling confident about self care and losing confidence in that and, therefore, needing to do something different?' **(Female, FG1)**
		Contextual barriers	'And then that turned into a chest infection and it was really bad. I don't normally call the doctors because I don't have the time as well because I work almost 13, 14 hours a day and by the time you come home the surgeries closed and telephone assistance is not that great with my surgery.' **(Female, FG4)**
Health-seeking behaviours	Memory attention and decision making; Belief about consequences; Environmental context; Emotions	Judging seriousness of symptoms	'Oh, if I'm getting aches and pains and generally starting to feel like I can't breathe or when you know it's affecting your ability to concentrate. Anything like that where I'm beginning, I'm really feeling this is not just an easy cold to manage. This could have quite an impact on other people, then that's the time that I [seek help], because I'm self employed as well. So it's a really hard judgement call but it is weighing it all up and at the end of the day I have to act in the best interests of the people that I'm treating.'<br>**(Female, FG3)**<br><br>'**Female participant 1**: I think I, I think for me a reasonable amount of time would be three weeks [to seek help from GP], I think beyond three weeks I'd tend to start thinking, oh God, what if I've got a chest infection? Or what if I've got something else?

		**Female participant 2**: I wouldn't have gone ten weeks because I think, oh God, what if you did have something really serious then you could have ten weeks you could have died. Do you know what I mean? I'm not being funny.<br>**Male participant:** Yeah, I kept, looking at, phlegm…and I was looking for blood and if it was blood, I would have gone straight [to seek help] because of the lungs, but it wasn't blood and I'm thinking, no, leave it a bit longer.'<br>**(FG2)**
	Deciding to seek help	'I think after COVID I start to get a bit more anxious now thinking is it something more serious and I think if I had more of a cold now, I'd probably seek more medical attention…' **(Female, FG5)**<br><br>'But I wouldn't know that. But for me it's like I'm not a frequent visitor to the doctor, so I'm not going to book, I don't want to waste lots of time, my time or their time, I'm very conscious, I would rather than appointment goes to someone who really needs it –'<br>**(Female, FG1)**
	Triaging and accessibility	'I wait at least for 20 minutes for your phone call to be answered and then the receptionist would say, oh I'll pass your message to the GP and she will call you between this time and then when you are working you can't answer the phone. And so it just, no good now.'<br>**(Female, FG4)**<br><br>'Yeah, I think I was a bit thrown when I was initially asked to provide my symptoms to a receptionist because I think I was probably just sat at my desk making an appointment at lunchtime, so I wasn't really prepared or in that environment…' (**Female FG1**)
	Children and elderly	'I'd be much more inclined to contact, to go to the pharmacist sooner and contact the GP, especially, my mother had dementia for five years and for three and a half of those was, she couldn't advocate for herself at all and you're worried about aspiration and all those things that are, so yeah, probably.' **(Female, FG1)**<br><br>'…with kids it's always a bit more worrying if they lose their appetite for days on end and they're not eating anything. And they can't always put into words what it is that's wrong. So I suppose with kids it's a bit different than with adults. And I think

			probably my tolerance for my kids being ill is a lot lower than my tolerance for myself being ill…' **(Female, FG3)**
		Community pharmacy	'I think sometimes it's just more convenient, it's like I went to the pharmacy recently to get my daughter some cough medicine and I wouldn't have made a doctor's appointment for her cough because she had only had the cough for four days but it was like on the fourth day it was keeping her up in the night' **(Female, FG2)**<br><br>**Female participant 1:** Because I think what I, historically to me what a pharmacist has been is when you know what you've got and you're going to ask what they can give you to make you feel better, what on your shelf is, can you give me?<br>**Female participant 2:** Yeah, so a salesperson rather than an expert.<br>**(FG1)**<br><br>'[a pharmacist] doesn't know your whole medical history which is, obviously the doctors have the overall picture of your body rather than a specific issue and that's where the doctors are paramount in having this position. And there's topics that you just want to discuss privately rather than to the high street.' **(Male, FG4)**
Healthcare expectations	Reinforcement; Goals; Knowledge; Belief about consequences;<br>• Intentions	Experience receiving infection advice	'Yeah, my youngest had a sting whilst he was on a Cub camp and they went to the local pharmacy and the pharmacy did suggest that they got a pen and drew round the sting site and when the red edge moved to the circle, that was the time he should be seeking a doctor's appointment or go into A&E and so it went very quickly.' **(Female, FG1)**<br><br>'I find my local surgery, if there's something that you're not necessarily familiar with that they've diagnosed, they're brilliant at, there and then in the appointment, they'll print off something, even if it's the Google description of it or they quite often use their own online practitioner site…So actually we could sit and read about it and then she was, she was more than happy with self-managing that at home, because we knew what it was…providing us the tools to actually then take home and deal with it.' **(Female, FG3)**
		Expectations for consultations	'Hopefully a way to end this illness. I don't want to come out thinking I'm none the wiser than what I was before. I'd like to know that there's an end in sight.' **(Male, FG3)**

		'I find that it's a bit embarrassing. If I'm speaking to a medical professional I'm probably looking for reassurance as much as a cure. I don't, I only tend to see someone if I'm feeling worried about myself because I'm not a very ill person, I'm a bit of an anxious person I have to like, I just want to be told, oh it's fine. You should be OK. It's not cancer it's a cold.' **(Female, FG4)**<br><br>**Interviewer: what kind of outcome are you looking for when you go and discuss your symptoms [at community pharmacy]?**<br>Female participant 1: Avoiding having to go to the doctor's surgery.<br>Female participant 2: Yeah, I agree, if you can avoid waiting and having to make an appointment or at least getting confirmation that you definitely should be seeing the GP, affirmation of your own gut feeling really.<br>**(FG5)**
	Beliefs about antibiotics	'…that belief that if you take antibiotics too frequently then they don't actually work as well. That's always been drummed into me, don't take antibiotics for everything'<br>**(Female, FG3)**<br><br>'**Female participant 1**: I know very little about [antibiotics] to be honest, only that the stuff in the media about having too much of it reduces its ability to work as well, so it's almost like a, taking it to me seems like a very negative thing.<br>**Female participant 2**: The best way of putting it, it's taking something away from you rather than empowering you, giving you something positive to know, yes.<br>**Male participant:** There's a message out there about take all of these because if you don't take them all you're leaving something around which may --Affect your family and other people, and that feels --<br>**Female participant 2:** Take the entire course and a lot of people want to come off the pills as soon as they feel well.'<br>**(FG1)**<br><br>'**Female participant**: Yeah, I guess you know if you've got symptoms of ear infection, tonsillitis, those sort of things where you know that you're going to need antibiotics to be able to get better.<br>**Male participant**: Oh definitely, yeah.'

	**(FG3)**
	'If I feel I have a urine infection actually I would take a sample and take it the doctors and I would expect antibiotics to be offered to me, to be honest. But that might be my lack of knowledge about urine infections.' **(Female, FG4)**

TDF – Theoretical Domains Framework. FG – Focus group.
