# Supplementary material for: Empowering Patients to Self-Manage Common Infections: Qualitative Study Informing the Development of an Evidence-Based Patient Information Leaflet
Source: Antibiotics (Basel). 2021 Sep 15;10(9):1113. doi: 10.3390/antibiotics10091113 (PMC8464808; doi:10.3390/antibiotics10091113)
Supplement: Supplementary file 1 [file antibiotics-10-01113-s001.zip › Table S2.pdf]

antibiotics

MDPI

*Article*

# Empowering Patients to Self-Manage Common Infections: Qualitative Study Informing the Development of an Evidence-based Patient Information Leaflet

**Catherine V. Hayes *, Bláthnaid Mahon, Eirwen Sides, Rosie Allison, Donna M. Lecky and Cliodna A. M. McNulty**

Primary Care and Interventions Unit, Public Health England, Gloucester GL1 1DQ, UK; Blathnaid.mahon@phe.gov.uk (B.M.); Eirwen.sides@phe.gov.uk (E.S.); Rosie.allison@phe.gov.uk (R.A.); Donna.lecky@phe.gov.uk (D.M.L.); Cliodna.mcnulty@phe.gov.uk (C.A.M.)

* Correspondence: Catherine.hayes@phe.gov.uk

**Table S2. HCP emerging themes on provision of advice on common infections (additional quotes).**

| Theme | TDF Domain(s) | Sub Themes | Quotes |
|---|---|---|---|
| Roles and responsibilities | Belief about capabilities; Optimism; Knowledge; Skills; Social and professional role | Motivation for AMS and promoting self-care | 'Optimistic, well I'm optimistic in terms of what I can do to educate the patients, whether than then translates into a reduction in resistance is another matter. But I'm certainly confident in what I do.' **(INT 4, GP, Male)**<br><br>'So I think there's lots of resources already available, but I think the main thing for me is always ensuring that the safety netting is there. With community pharmacists if you have, if you supply a regular prescription you get to know the people and in the next few months there's going to be a new service where as soon as a patient is discharged from hospital the discharge information will go to the community chemist that is going to continue looking after the patient, as well as the GP surgery, is the plan.' **(FG1, pharmacist, female)** |
| | | Professional responsibilities | 'If you have counter assistants… their role is to give basic advice and then the more complicated things should be referred on to a at least a technician… So, I think it's about each staff member knowing their limitations and knowing when to give out advice and that they know what that advice is and knowing when to refer back to the pharmacist. But then also it's incumbent upon the pharmacist to know their limits as well and to recognise the need to refer on further if needed.' **(INT 1, pharmacist, male)** |
| Approaches to managing common infections | Belief about consequences; Belief about capabilities; Goals Social influences; Environmental context | Importance of self-care advice | 'I personally place a huge emphasis on self-care because in this day and age of consent, shared decision making, empowering the patient as well, culturally we've moved away from being told by healthcare professionals what to do and how to do it exactly. It is very much a collaborative process' **(FG1, pharmacist, female)** |
| | | Shared decision making | 'So I think the first thing is we should be trying to identify what is the expectation of the patients, sometimes we don't allow time for that and I think it's then very much going down that shared decision making route. And trying to strongly counsel against antibiotics and saying, giving people defined periods of time within which the infection is likely to get better of its own accord. And then also safety netting is very important, so if things worsen or don't get better within a period of time, then you would like to see that patient again.' **(FG1, GP, male)** |

| | | | |
|---|---|---|---|
| | | Encouraging use of community pharmacy | 'Yeah, I also encourage people to go to their pharmacists more now. I think that's going to be a really important message to get out to people is that, it should be that your first port of call is to go and see a pharmacist, I know that's not a very specific thing about, but and they have a whole range of OTC self remedies if you like and symptomatic control. I don't, I think that's another important thing is trying to get, get away from the expectation that just coming into the doctor because it's, the prescription's free, if we can try and encourage people away from that, that's a useful thing. ' **(FG1, GP, male)** |
| | | Approaches to educating patients | 'I think the really brief description of the evidence that antibiotics don't work for X, Y, Z, they don't work for viruses, they don't work for stomach bugs and I think what really helps is, not only telling them that they don't work, but that these drugs also cause many side effects. So, I think, when you mention to people that they can cause more stomach upset or more nausea, more vomiting, and with certain types as well, like it's known that they can affect *your neck,* they can affect tendons. I think people have a perception that antibiotics are a benign type, benign class of drugs plus, I think, if people are made more aware of the side effects, it'd probably help with the interaction.' **(INT 1, pharmacist, male)** |
| | | Variations in practice and lack of monitoring | '[about remote consultations] …can't physically assess their illnesses and [GPs] will probably prescribe more than if they were able to have that face to face physical assessment just to err on the side of caution unfortunately.' **(FG1, pharmacist, female)**<br><br>'I think it's really difficult, I worked in a practice which had six partners plus extra doctors and the variation in the threshold for prescribing was enormous. Absolutely enormous, shocking really. And I think if you've got that variation at a clinician level it's very difficult to expect staff to have consistent messaging.' (**FG1, GP, Male)** |
| Patient attitudes and context | Belief about consequences; Belief about capabilities; Environmental context; Social influences | Beliefs about patient attitude/expectations | 'Patient attitude is definitely one thing as well because it can be quite clear the patients might have a pathway in their mind about what the treatment should be like, for instance, rather than taking the information on board. I think that's probably the biggest barrier, patient expectation.' (**INT 1, pharmacist, Male)** |

| | | |
|---|---|---|
| | Beliefs about effect on patient behaviour | Oh, it's difficult to tell really. I tend to, I think the one thing that they will do is possibly take the time to rest if I've formally given them, as an authority figure, the permission to have a day off, particularly the younger ones. Whether people will actually take the shopping list of self care advice away, well mostly they do, to be absolutely honest. It's the way you present it really. Of course, you ultimately don't know, but if they take it away with a smile and pocket it, as opposed to leaving it on the table and, that's generally a sign that they've actually taken it away and popped it in their purse or their pocket, if you know what I mean? **(INT 2, nurse practitioner, female)** |
| | Mass media / public health campaigns | 'I think we could probably do more to promote self care, I think we could perhaps maybe adverts in newspapers, we could have more of a social media footprint, I think there is probably more that we can do in terms of promotion.' (**FG1, pharmacist, female)** |
| | Patient contextual barriers | 'But the economics of it is often quite difficult. I have prescribed paracetamol in families that I know would have struggled… but you have to bear in mind that if they really are struggling financially, all the self-care advice that you give is going to be difficult if they can't afford it.' **(INT 2, nurse practitioner, Female)** |

**Table .** DF – Theoretical Domains Framework. FG – Focus group. INT – Interview. GP – General Practitioner.
